# Dr. Paul F. Bussman

**Published:** 1913-01

**Authors:** 


Dr. Paul F. Bussman, Buffalo, 1882, of Buffalo, died Dec. 19, aged 52.

